# Ortho2ExpressMatrix—a web server that interprets cross-species gene expression data by gene family information

**DOI:** 10.1186/1471-2164-12-483

**Published:** 2011-10-04

**Authors:** Thomas Meinel, Michal R Schweiger, Andreas H Ludewig, Ramu Chenna, Sylvia Krobitsch, Ralf Herwig

**Affiliations:** 1 Structural Bioinformatics Group, Institute for Physiology, Charité - University Medicine Berlin, Thielallee 71, 14195 Berlin, Germany; 2 Vertebrate Genomics Department, Max Planck Institute for Molecular Genetics, Ihnestrasse 63-73, 14195 Berlin, Germany; 3 Institute of Human Nutrition and Food Science, Christian-Albrechts-University of Kiel, Heinrich-Hecht-Platz 10, 24118 Kiel, Germany; 4 Biotechnology Center, Technical University Dresden, Tatzberg 47-49, 01307 Dresden, Germany; 5 Otto Warburg Laboratories, Max Planck Institute for Molecular Genetics, Ihnestrasse 63-73, 14195 Berlin, Germany

## Abstract

**Background:**

The study of gene families is pivotal for the understanding of gene evolution across different organisms and such phylogenetic background is often used to infer biochemical functions of genes. Modern high-throughput experiments offer the possibility to analyze the entire transcriptome of an organism; however, it is often difficult to deduct functional information from that data.

**Results:**

To improve functional interpretation of gene expression we introduce Ortho2ExpressMatrix, a novel tool that integrates complex gene family information, computed from sequence similarity, with comparative gene expression profiles of two pre-selected biological objects: gene families are displayed with two-dimensional matrices. Parameters of the tool are object type (two organisms, two individuals, two tissues, etc.), type of computational gene family inference, experimental meta-data, microarray platform, gene annotation level and genome build. Family information in Ortho2ExpressMatrix bases on computationally different protein family approaches such as EnsemblCompara, InParanoid, SYSTERS and Ensembl Family. Currently, respective all-against-all associations are available for five species: human, mouse, worm, fruit fly and yeast. Additionally, microRNA expression can be examined with respect to miRBase or TargetScan families. The visualization, which is typical for Ortho2ExpressMatrix, is performed as matrix view that displays functional traits of genes (differential expression) as well as sequence similarity of protein family members (BLAST e-values) in colour codes. Such translations are intended to facilitate the user's perception of the research object.

**Conclusions:**

Ortho2ExpressMatrix integrates gene family information with genome-wide expression data in order to enhance functional interpretation of high-throughput analyses on diseases, environmental factors, or genetic modification or compound treatment experiments. The tool explores differential gene expression in the light of orthology, paralogy and structure of gene families up to the point of ambiguity analyses. Results can be used for filtering and prioritization in functional genomic, biomedical and systems biology applications. The web server is freely accessible at http://bioinf-data.charite.de/o2em/cgi-bin/o2em.pl.

## Background

Development and progression of diseases are to a large amount linked to evolutionary conserved genes [1] as well as to reactions on environmental factors such as infection or nutrition. Many experimental model organisms including mouse, worm, fruit fly or yeast were established to study such factors when human material could not sufficiently be exploited. Such developments necessitate tracing the descendents of common ancestor genes in these organisms where gene conservation is typically judged by sequence similarity. Genes similar in sequence can be found not only in different organisms but also in a single organism after the appearance of evolutionary events such as genome duplications or the invention of alternative splicing isoforms (ASIs).

Today's attempts to systems biology integrate experimental insights from wet labs combining the fields of functional genomics, biomedicine and bioinformatics. The *function *of genes is either determined experimentally or predicted by similarity of respective sequences. In the same way, sequence similarity is commonly regarded as a valid measure for gene *evolution*. Hence, the conservation of gene sequences as well as of gene function refers to a common origin but this conservation does not exclude that gene function probably changes [2-5]. On the other hand, gene function can be indirectly derived from gene expression analyses regarding expression profiles as reactions patterns. Several aspects such as the essentiality of homologous genes [6] in normal and disease networks [7] are studied quantitatively using genome-wide gene, transcript or protein expression data. Hence, it would be beneficial to infer gene function with an integrated approach combining sequences and quantitative measurements.

The basis for such cross-species comparisons is the precise determination of orthology relationships between full-length protein sequences. Current family inferring approaches are aiming at the computation of the closest proteins of two organisms, the *orthologs*, or of a single organism, the *paralogs*, but the approaches are generally not capable to discriminate molecular individuals of similar functionality among a set of similar genes, e.g. within a single organism [8,9]. Such approaches have been analysed and compared [10]; essentially, these approaches consist of a sequence similarity comparison with tools like BLAST [11] along with specific categorization algorithms and processing pipelines that exploit the results of the sequence similarity. Sets resulting from such categorizations are named orthologs clusters or protein families; the latter term is used for all such results in this paper. Depending on the specific algorithm for determining protein families, either a reduced [12-14] or the completely available organism range [15] is used. Strategies of the implementations aim at performing a strictly pairwise organism comparison [16,17], large-scale comparisons for multiple species [15,18], robust clustering algorithms in uncurated pipelines [15,19], or the algorithm pipelines include reconciliations or further phylogenetic data [13]. During the last two decades, a large amount of such approaches were created [10,20] and it is often difficult to retrace the results of such procedures. Respective repositories were established as searchable databases and are, currently, the ends of the information processing queue. In the context of quantitative analytics or systems biology it is desired that such successful and excellent information resources are used, for example as components of integrative data pipelines. As well, comparisons across particular established family sets by a single-standing or integrative application tool are sparse.

Because of the complexity of evolution it becomes necessary to broaden gene expression analyses to the full set of paralogous genes. Current gene expression studies are able to quantify expressed genes in a genome-wide manner using microarray chip technology and, more recently, quantitative high-throughput sequencing. One problem of microarray analyses is that expression data are often compressed to gene level annotation, which does not reflect alternatively spliced isoforms. It is important to note that large repositories for microarray experiments, the Gene Expression Omnibus GEO, ArrayExpress, or the Gene Expression ATLAS [21-23], store, beside the raw data, already normalized data. Such resources are used to retrieve or derive differential expression data of published research on immune response, ageing, nutrition, compound or drug treatment, or experimental gene manipulations such as knock-out or gene silencing. There already exist helper tools [24] that facilitate cross-species queries in such repositories for finding ortholog relationships.

In this work we describe the web server Ortho2ExpressMatrix (O2EM) that integrates evolutionary information on gene (protein or microRNA) families and whole-genome expression data in order to i) compare the experimentally comparable settings for two biological objects; ii) find functional orthologs in a pair of species; iii) help to infer a meaningful sub-clustering of large gene families by gene expression data; and, iv) disclose a row of similarly differentially expressed genes in the light of sequence similarity. The tool represents a systematic approach for genome and proteome research and aims to contribute to functional orthology detection.

## Construction and context

### Organisms

O2EM requires background data for each organism including the entire protein family information and gene annotation cascade from gene names to protein identifiers. The current organism space for O2EM comprises H. sapiens (Hs; human), M. musculus (Mm; mouse), D. melanogaster (Dm; fruit fly), C. elegans (Ce; worm), S. cerevisiae (Sc; yeast).

### Gene annotation

Basic gene and protein information was retrieved from Ensembl [25] using the BioMart [26] service. The download includes gene names, descriptions and UniProt protein identifiers [27], separately for the four Ensembl releases 63 (of June 2011), 59 (of August 2010), 53 (of March 2009) and 49 (of March 2008). This variability opens the user the opportunity to retrieve results for a previous genome build, e.g. NCBI37 for human or to compare results across different genome builds (genome build versions for the five organisms and respective Ensembl versions can be found in Additional file 1, Table S1). Annotation of worm protein sequences was adjusted using the WormBase [28] for the sake of conformity with Ensembl.

### Protein family approaches

We retrieved publicly available data of four different protein family approaches (EnsemblCompara, InParanoid, SYSTERS, Ensembl Family) and integrated them into O2EM conserving the original associations. This selection of protein family approaches was based on coverage of the pre-defined organism space, diversity in general methodology (multi-organism and pairwise organism approaches), varying granularity of the families and acceptance by the community. We generated family data sets for pairs of organisms in a common file format. Additionally, we created data sets for paralogs to be able to compare two individuals from the same species. For all approaches it can be observed that family or cluster IDs are generally not stable beyond a particular version.

#### EnsemblCompara

Ensembl provides orthology relationships according to the EnsemblCompara method [13] in BioMart generally for pairs of species. Association data were retrieved for all organisms in the given organism space twice, in the forward way (querying orthologs of organism 2 by organism 1) as well as in the reciprocal way. Because EnsemblCompara does not provide own families or family identifiers, we determined connected components of orthologs integrating relationships of both directions. We generated unique and disjunct families out of the results and introduced a default numbering system. We additionally retrieved and generated separate paralog families for each of the organisms; the family identification was introduced analogously. However, only multi-protein families (one-to-many or many-to-many) were available; hence, all singleton paralog families must be ignored in O2EM.

#### InParanoid

The InParanoid database [16,17] is aimed to restrictively infer clusters of orthologous genes between two organisms [29]. InParanoid data are therefore available in O2EM only for cross-species queries. We included the InParanoid6 data set for Ensembl version 49 and InParanoid7 for Ensembl version 59 and above. Mouse and worm proteins identifiers were converted to standard Ensembl IDs where necessary.

#### SYSTERS

The SYSTERS set of protein families [30] for multiple organisms was parsed to retrieve protein sequence identifiers and SYSTERS family identifiers for pairs of organism as well as for single organisms in the given organism space. Because SYSTERS [15] mainly refers to the UniProt/Swiss-Prot/TrEMBL nomenclature, respective identifiers were converted to Ensembl protein IDs for each Swiss-Prot accession number (AC) and, if no conflict occurred, for each related TrEMBL AC. Conflicts can arise if a TrEMBL AC reflects a fragmentary sequence, which is then (by Ensembl/BioMart) associated with multiple genes and therefore could be assigned to multiple SYSTERS families. Hence, ambiguity was prevented because protein sequences are in SYSTERS methodologically associated to disjunct families. The update of SYSTERS release 5 is based on UniProt release 12.4 and was integrated into O2EM.

#### Ensembl Family

Families are generated for similar protein sequences [12] over a large set of organisms using the Markov Clustering algorithm [19]. This procedure is performed separately for each Ensembl version, introducing non-stable identifiers in early Ensembl versions, whereas newer Ensembl versions reflect the family generation in identifiers. We retrieved Ensembl Family sets and identifications separately for each of the three Ensembl versions. Subsets of respective protein families were established by filtering subsets of sequences separately for each pair of organisms (orthologs) and, additionally for each single organism, subsets of multiple sequences from single families (paralogs).

### MicroRNA approaches

The two family approaches for microRNAs, miRBase [31] and TargetScan [32], reflect either sequence similarities of microRNAs or the association of microRNAs to a shared set of target genes. Two sequence forms of microRNAs are systematically maintained by miRBase, which was selected as the reference resource for O2EM: the hairpin sequence, which is the precursor microRNA, and the mature microRNA, which is biologically processed from the hairpin and the regulating molecule. MicroRNA data sets are available for human, mouse, worm and fruit fly.

#### miRBase

The miRBase provides microRNA gene families as sets of similar microRNA sequences across multiple organisms. We excerpted associations of mature microRNAs to microRNA families and pre-defined miRBase family sets for each pair of organisms in the organism space. We also added respective star microRNAs to miRBase family sets to take a putative functional impact of star microRNAs into account.

#### TargetScan

We retrieved microRNA family associations from the TargetScan sets of human and mouse (TargetScan release 5.1) as well as of worm and fly from TargetScanWorm 5.1 and TargetScanFly 5.1. The mature and hairpin association in TargetScan refers to miRBase. In contrast to the data extraction for that resource, star microRNAs are ignored in O2EM's TargetScan families.

### Microarray platforms and probe set information

A number of prominent microarray platforms both for mRNA and microRNA analysis of the manufacturers Affymetrix, Illumina and Agilent are recorded in O2EM. The actual list of supported platforms—together with the corresponding GEO platform identification—is shown separately (Additional file 1, Table S2 and in the web tool's FAQs page). Associations of probe set identifiers to Ensembl transcript, protein or gene identifiers were retrieved from the BioMart service of Ensembl. They were not filtered according to ambiguity in any direction and were generated for the Ensembl versions 49, 53, 59, and 63 separately.

### Similarity measures between protein sequences

Reciprocal best hits were derived from pairwise all-against-all BLAST queries for each pair of organisms. Respective BLAST e-values were collected for all family members to characterize all pairs of the constituents of a protein family. However, it should be noted that O2EM families can not be re-computed with the tool since it uses already existing gene families according to the established approaches. Because BLAST e-values depend on the sequence length and the size of the reference database, they can differ significantly from family to family but are a good measure for the similarity between the homologs within a single protein family.

## Implementation

### Data input

The two organisms under analysis need to be pre-selected either by defining two (organism-specific) microarray platforms or directly by the organism names if data are associated to gene annotations (genes, transcripts or proteins). Data must be loadable in tab-delimited file format from the user's local computer. The tool accepts separate expression values for the two organisms to be compared (which also can be the same species) of both the treatment and the control experiments (i.e., as four plain text columns in total). Alternatively, pre-calculated ratios can be loaded (in two columns for each organism; options: ratios, log2-ratios, log10-ratios). Data may be given in one or two separate files; examples for data import are provided and the usage is described in the web tool. Annotation information can be given in one shared column or two separate columns for each organism. Input data can be assigned to probe set identifiers—a row of microarray platforms is integrated into O2EM and the association is then automatically performed—or to Ensembl gene, transcript or protein identifiers. Additionally, a separate microRNA annotation category is available beside respective microRNA-specific probe sets of several manufacturers.

Prior to starting the query and as an alternative to scrolling through the complete query result of all available protein families, the user can reduce the O2EM display to gene families of interest. Family identifiers must then correspond to those of the selected protein or microRNA family approach, or, if a list of genes is given, the display is reduced to respective (full) families. Furthermore, singleton families can be excluded which enables the user to set the research focus on either multi-gene families (i.e., two and more paralogous genes for at least one organism) or multi-protein families (two or more ASIs).

### Pre-selection criteria

O2EM supports the pre-selection of the output result according to significance of gene expression. The tool performs an automatic pre-selection and data filtering for significant values as well as for criteria of co-direction in both organisms; O2EM exploits HTML checkboxes for both purposes. Significance of differential expression in O2EM is judged by a fold-change threshold (default is < 3/4 or > 4/3) which can be set by the user. Co-directed expression, i.e., identical direction of fold-changes in the two organisms, can be used as an important data filtering criterion: co-directed over expression only, co-directed repression only, both together, both anti-directed directions (under-over and over-under expressions). The pre-selection enforces the automatic check-in of HTML check boxes which the user can manipulate after the pre-selection. As option of the pre-selection, the tool generates a plain text ASCII output in the subsequent step.

### Operations during the procedure

**Figure 1:**
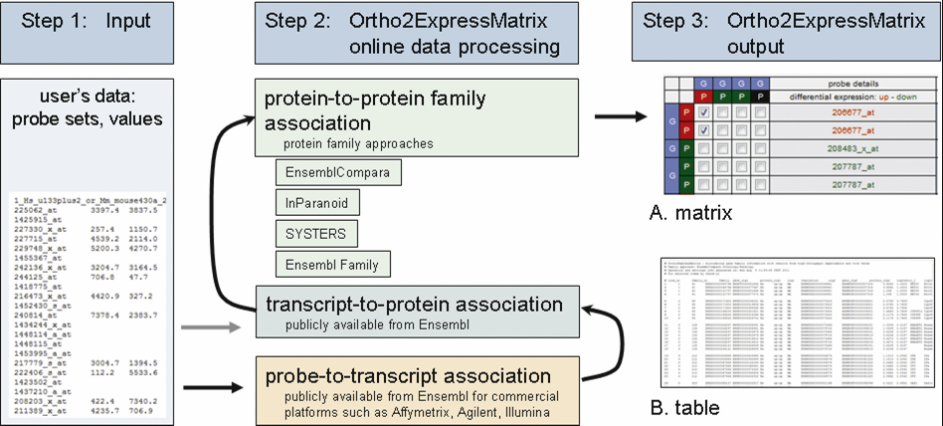
**Ortho2ExpressMatrix workflow and usage**. Several different formats of the user's data are accepted by the web tool that allows the entry at several stages of the processing pipeline (step 1, input). Input data comprise either expression values for treatment and control or respective expression ratios of each of the two organisms or individuals to be compared. The processing, shown in step 2, covers the gene annotation levels from probe sets across transcript, protein to protein family annotation. Here, several external sources are named such as EnsemblCompara, InParanoid, SYSTERS, Ensembl Families for protein family approaches. The other annotation associations are derived, mainly from Ensembl, for the five species human, mouse, fruit fly, worm and yeast. Step 3 symbolizes the web server output (A) as matrix visualization and (B) as table. Similarly to the shown protein family association, the tool performs the association task also for family information of microRNAs.

The data processing pipeline (Figure 1) comprises the automatic annotation from a given probe set to the transcript, and from the transcript to the respective protein as well as back to the gene. O2EM automatically performs the ratio calculation of differential expression if expression values for treatment and control are imported. Differential expression of all transcripts in a family is always re-calculated to log2-ratios and is colour-coded. Family members without expression values are automatically excluded from the matrix display. Ambiguous probe-to-gene or probe-to-transcript annotation does not lead to exclusion of respective probes to elucidate ambiguity. Expression values of redundant probes are averaged (arithmetic mean) in the colour boxes. Additionally, single probe sets and values assigned to each single transcript are displayed in a separate column to identify ambiguity and divergent expression directions of particular probe sets. BLAST e-values indicating similarity for each protein sequence pair are loaded from the tool's data background.

### Statistical analysis

O2EM allows over-representation analyses (ORA) based on Fisher's exact test [33] to validate the significance of the expression status for each protein family separately and for each organism. This analysis compares the list of differentially expressed genes and assesses for each family whether an over-representation is observed. As population background O2EM uses the number of all genes. In O2EM, significant differential expression is summarized according to gene annotation based on probe sets, genes, transcripts or protein levels as it is selected by the user on the tool's entry page. Consequently in O2EM, each level can be used for the ORA according to the user's definition.

The mathematical background of the ORA is the hypergeometric distribution which is implemented in O2EM comparably to the function 'phyper' of the R package. The procedure in O2EM regards the ratio selected in the entry page as significance threshold. Separate calculations are performed for each of the two organisms (the objects along the two axes of a matrix) as well as for higher and lower differential expression. Hence, the results comprise four p-values for each family.

## Utility

### Online matrix visualization

O2EM is designed to give the user full control—and responsibility—over input and quality of expression data assuming that the data for upload into O2EM are already normalized and summarized to a single expression value for each feature (e.g. median or average expression value associated to any gene annotation). The intention of O2EM is that the user shall be free to combine data levels of annotation and expression values with one out of several different gene family approaches.

**Figure 2:**
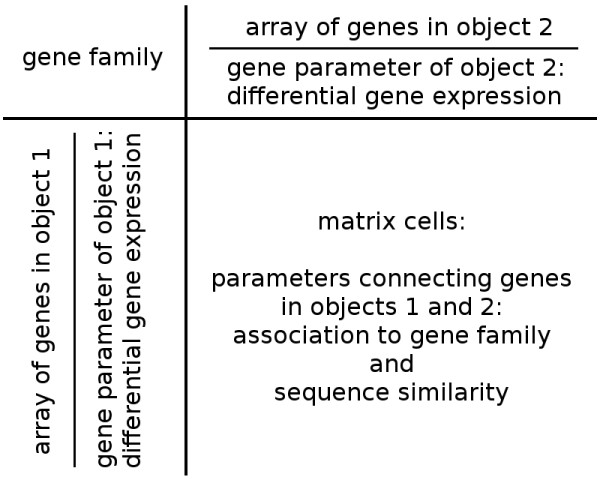
**Information content of gene family matrix view**. The two biological objects 1 and 2 (organisms, individuals, see text) that are influenced by a disturbing factor (disease, treatment, experimental setting, see text) are placed along the axes together with corresponding arrays of gene annotation and the first gene parameter, the 'differential gene expression'. The second gene parameter 'sequence similarity' connects all members of a single protein family in the matrix field of the visualization.

O2EM visualizes each single gene family in a matrix format. Figure 2 illustrates the schema of the O2EM matrix display in the web. All members of a single gene family are assigned to the row and the column of the matrix. The assignments for row and column with respect to the two biological objects include the gene annotation and the information on differential gene expression. Each single cell in the matrix symbolizes the membership in the gene family which is connected with the similarity of the two joined sequences; such a sequence pair can also be selected for data display with a HTML check box.

O2EM matrix displays of gene families provide intuitively insights into experimental gene expression and sequence similarity data. The two dimensions of the matrix indicate simultaneously (i) the sequence identifiers—cursor mouse-over function and URL link to the data origin and thereby external information on gene function—and (ii) the expression values as colour-coded fields in three grades: green/black/red for the directionality of differential gene expression (lower/not significant/upper). Averaged differential expression values are presented by curser mouse-over. Each cell of the matrix is coloured representing the sequence similarity of the respective pair of protein sequences; for this purpose, BLAST e-values are translated into colours; the colour code is explained in the web tool's FAQs page. The respective BLAST e-value is visible by cursor mouse-over or in the table output (see below).

Furthermore, additional information is given for genes in the vertical axis: each gene is linked to the source database (i.e., Ensembl) and, additionally, to the KEGG database [34] in order to explore gene functionality in signalling and metabolic pathways. A further link to the ATLAS database [23] is provided to give the user the opportunity to explore similar gene expression data in foreign studies for the respective gene (in a particular row) as well as for the full set of orthologous genes (all columns) in the compared organism. Matrices can be transposed if the orders of input organisms as well as of the data files are mutually changed. The genome-wide set of gene family matrices is continuously displayed. Scrolling through batches of 50 consecutively displayed families provides genome-wide insight into differential gene expression across gene families.

### Reports on data loss

Data loss occurs in several ways and depends on the data configuration: (i) if proteins are not assigned to a family in a particular family approach; (ii) if transcripts encode non-protein coding genes and a protein family approach is selected; (iii) if gene sequences are not covered by a (microarray) platform; (iv) if significance criteria, as defined by the user, are not satisfied. However, data quality criteria are in the user's responsibility and data will not be validated by the tool (e.g., if probe signals are not distinguished from the background; 'absent calls'). Information on complete data reduction is given for several mapping steps at the end of the O2EM result pages.

### Table output

Three table output options are available in addition to the matrix view. The pre-selection—HTML check boxes allow (i) for a table view of the selection content shown on the actual matrix result page; furthermore, (ii) the entire result set as well as (iii) the set of all relations of significant ratios can be retrieved either from the entry page or from each single matrix result page, independently from the actually displayed batch. The output comprises information on family, gene, protein (or mature microRNA, respectively), the differential expression as log2-ratio for each of the two organisms as well as the BLAST e-value for each protein sequence pair; the format is tab-delimited ASCII plain text.

### Case study

In order to exemplify the usability and capability of O2EM, and also to clarify technical and information utilities of O2EM we conducted a case study. To elucidate connections within single families, we take two example families with co-directed expression from the literature. The first example comprises *RUNX1 *genes in human as well as in mice and further differential expression data of *RUNX1 *paralogs (*RUNX2, RUNX3*). Additionally to the *RUNX *family, we present a second protein family that is reported to be functionally connected to *RUNX1 *depletion.

Silencing or knock-out studies with the human transcription factor *RUNX1 *have been performed using the human megakaryoblastic leukaemia cell line Meg-01 [35] as well as juvenile mice [36]. As reaction on the *RUNX1 *knock down, a significantly decreased gene expression is reported for the delta polypeptide of the catalytic Phosphoinositide-3-kinase, *PIK3CD*, a member in the human *PI3K *family. Similar genome-wide experiments explored reactions of *Pik3cd *on *Runx1 *silencing in somatic cells of juvenile mice. We retrieved resulting gene expression data, series GSE17311 and GSE2592, from the GEO repository [21].

**Figure 3:**
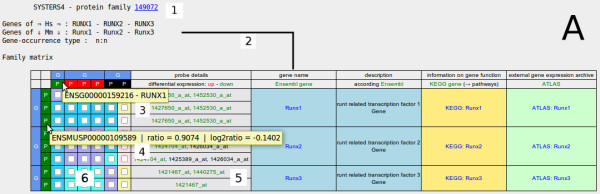
**Ortho2ExpressMatrix case study A**. *RUNX1 *data were extracted from two different public resources. Data in GEO series GSE17311 (related literature, see text) include log10-ratios of two channel differential expression data in four experiments using the human megakaryoblastic leukaemia cell line Meg-01; GSE2592 comprises expression data for *Runx1 *knock-out and wild-type mice at two time points and for all conditions done in duplicate. We extracted particular experiments from both data series for comparison with O2EM. Data for the human cell line were prepared for upload into O2EM by pre-calculation of the median from four biological replicates (combining data of biological or technical replicates are always in the user's control). Mouse data were separately averaged for the four knock-out and for the four wild-type experiments. The annotation function of O2EM associates probe information to gene and protein identifiers (transcripts and proteins stand, according to Ensembl, in a 1-to-1 correlation; transcript annotation is generally ignored in O2EM matrices) and finally to the families. This Figure, SYSTERS protein family 149072, in detail: annotation for *RUNX *genes of mouse (number 1) is displayed on the vertical axis, human orthologs are displayed on the horizontal axis. Gene names including links to Ensembl and gene description (near number 2) are located in the blue fields (additional links to KEGG genes and ATLAS experiments are truncated). The two items of the matrix borders comprise gene annotations in outer sphere (number 3) and, in the inner sphere (number 4), expression values of transcripts and information on related proteins; log2-ratios of differential expression intensities are averaged across all probe sets and translated to red/green colours (see section: Utility/Online visualization). Additionally, identifiers and intensities for each single probe set are given in a separate column (number 5; mouse-over). Background colours of the matrix cells (number 6) indicate similarity of the protein sequence pairs in the respective columns and rows. The reader should note the violet cell background that indicates the most similar proteins and respective genes and hence the respective closest orthologs, here distinguishing *RUNX1-Runx1 *genes from *RUNX2-Runx2 *and *RUNX3-Runx3 *genes and vice versa.

**Figure 4:**
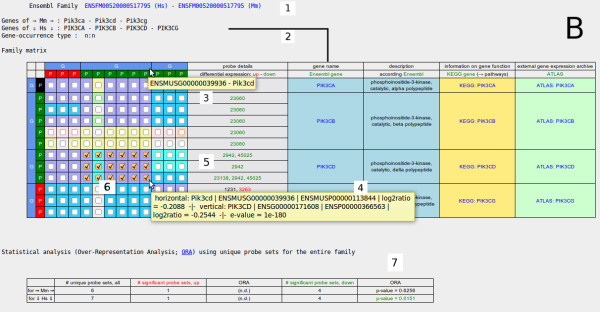
**Ortho2ExpressMatrix case study B**. *PIK3CD *data were extracted from two different public resources, confer Figure 3. This Figure shows the respective Ensembl Family ENSFM00520000517795 for *PIK3 *genes (alpha to delta subunits of the catalytic phosphoinositide-3-kinase) of human and mouse; the reader should note that the organism configuration is mutual to Figure 3. Explanations in numbers 1, 2, 3, 5 and 6 correspond to the numbers in Figure 3. Pre-selection such as 'lower differential expression parallel in both organisms' enforces the automatic check-in of the HTML check boxes and the mouse-over explanation as shown (the lower differential expression of *PIK3CD *in both organisms as reaction on RUNX1 silencing/depletion is the focus of the original literature, see text). This Figure shows a manually reduced pre-selection that shall indicate that there are now three proteins (ASIs) of the human gene *PIK3CD *and six proteins (ASIs) of the mouse gene *Pik3cd *selected for the Table output display (number 4; see also 'Utility/Table output'). The statistical analysis (number 7) shows significances given by down-regulated probe sets and calculated by over-representation analysis p-values in mouse and human.

Figure 3 and Figure 4 show two O2EM matrices for the respective two protein families in order to provide family information on both organisms (by calling two alternative protein family approaches). The paralogous sequences of *RUNX1/Runx1 *(Figure 3; SYSTERS4 family 149072) as well as of *PIK3CD/Pik3cd *(Figure 4; Ensembl Family Ensembl:ENSFM00520000517795) were taken as screen shots from the O2EM output displays. The *RUNX1/Runx1 *knock-out/silencing can be traced by a lowered expression in human knock-out cells (indicated by green boxes near the Ensembl ID ENSG00000159216) as well as in mouse (background of the box for Ensembl ID ENSMUSP00000109589). In mouse knock-out animals, expressions of all other *Runx *genes were also lowered while in human *RUNX2 *was over-expressed and *RUNX3 *was not significantly expressed. Such different co-directional behaviours discriminate the genes in the *RUNX *family from each other. In agreement with Edwards et al. [35], the genes *PIK3CD *and *Pik3cd *are repressed after the knock-out of *RUNX1/Runx1*, which could be quickly traced in the corresponding O2EM matrix, see green boxes at the matrix borders and the check box ticks in the matrix cells. Relationships of corresponding paralogs in both organisms can be easily traced by the coloured matrix cells in Figure 3 and Figure 4.

## Discussion

In order to exemplify the biological complexity covered by O2EM, we discuss the main features in the sequel.

### Selection features

O2EM features variability in: technology of measurements (microarray, microarray two-channel, quantitative next generation sequencing, etc.); microarray platforms (more than thirty platforms; see the web tool's FAQs page); homology relationship for orthology studies across two species (within the current organism space) or across paralog families of single species (tissue, technology comparison); gene family approaches (currently four for proteins and two for microRNAs); gene annotation levels if input data (probe set, gene, transcript, protein); form of differential expression data (two values to be calculated, ratio, log2-ratio, log10-ratio); actuality of the transcriptomes according Ensembl versions 63, 59, 53 and 49.

### Interpretation of the matrix

O2EM is open for investigations in both directions: finding similar genes and discriminating them by differential gene expression, for instance, finding candidates of functional orthologs for two different organisms—inside-to-outside of the matrix—; or discriminating similar gene expression of transcripts within a protein family by sequence similarity—outside-to-inside. None of the directions overrules the other; moreover, both information trajectories contribute to a more complete picture.

### Ambiguity

The steadily increasing number of transcript annotations for a single gene reflects the continuous progress in the development of ASI prediction algorithms. It ends up also in an increase in ambiguity of probe sequence associations to transcripts since microarray designs are fixed. O2EM additionally favours transcript-associated expression data and, thus, circumvents ambiguity problems of the probe set annotation level. Coping with the transcript annotation level O2EM supports also more recent technologies such as next generation sequencing. Even ambiguity of probes on gene level is traceable; examples are genes in families of gene of the major histocompatibility complex.

### Family approach benchmarking

The functionality of the O2EM web server is enabled by several pre-generated data that are stored in the tool's data background. For instance, the tool holds the proteins as members of established protein family approaches, and the total number of stored proteins and protein families varies for each pair of organisms and for each single protein family approach. We counted such numbers to provide an overview over content and granularity of the single approaches. Comparably, Ensembl version 59 (Additional file 1, Table S3) and Ensembl version 49 (Additional file 1, Table S4) show protein numbers in families as well as numbers of protein families for organism pairs and single organisms. The increase in protein numbers between Ensembl versions 49 and 59 reflects the improvements of splicing isoform detection by respective algorithms. A similar compilation can be found in [13].

### Established protein family inferences

Established protein family inferences of all in O2EM integrated (external) approaches possess often limitations in the analysis of a sequence comparison [10], the evaluation step (clustering, phylogenomic analysis, etc.). O2EM attacks the problem indirectly by the implementation of methodologically different family approaches. This makes family inference results biologically more comprehensible since the approaches can be indirectly compared by successively running a discrete data set through all implemented family approaches.

In O2EM, all protein family data sets are based on full protein sequence length. In contrast to data of those established approaches (each of them is characterized by the evaluation step), pure BLAST searches could induce misleading results since BLAST best hit results, evaluated by e-values, are often driven by ubiquitary domains. Hence, the whole sequence length definition of the family generally overrules also a protein domain family definition, as for example provided by Pfam [37], and moreover a single best hit between two sequences. This is a strong argument for an integrating tool like O2EM that utilizes established protein family inferences based on full sequence lengths.

In O2EM, the user better exploits larger families (SYSTERS, Ensembl Family) to study the set of paralogs or finer granular families (EnsemblCompara, InParanoid) for the detection of the closer orthologs. Sequence relationships within larger protein families can be explored by the matrix visualization through colours that are coding sequence similarity. This feature facilitates and expedites visual analyses of lots of gene families.

### Candidates of functional orthology

O2EM with its attributes as an exploration tool bears forthcoming aspects for potential research. Generally, a sub-clustering of large families should be successful using differential expression data. This hint shall indicate that such principal observations can be made with O2EM. This can directly focus into the detection of candidates of functional orthology, which concerns the functional identity of an orthologous pair of proteins in two species. Expression traits of orthologous genes should be similar if such proteins stand in a functional context of pathways or protein complexes. Strength of O2EM is that common functional contexts can be compared with particular similarities of the proteins in a family.

### MicroRNA families

Similar to protein coding genes, also microRNA families can be analyzed due to their activation status. Results are displayed in Additional file 1, Figure S5 for the two implemented microRNA family approaches miRBase and TargetScan simulated with random data for two patients. The Figure highlights the visualization of gene identity, pre-selection of co-directional expression, star microRNAs and family size. Members of microRNA families show a high sequence similarity which is a hint at common targets (a strong argument of the TargetScan approach); and moreover, O2EM provides insights in the regulation of such microRNAs. - As a further filtering function, the variation of the significance criterion (Additional file 1, Figure S6) can be used to set the focus on stronger activated or deactivated microRNAs. - To elucidate the genetics of several diseases it is an eminent research topic to explore the expression status not only of microRNAs but also of the potential target mRNA. Studying both RNA sets together makes O2EM to a basic application in functional genomics.

### Paralog-rich protein families

Such families consist of genes with common evolutionary background, such as genome duplication before or after speciation, or with qualities that require high functional specificity such as binding specificity of genes in the major histocompatibility complex MHC. Cancer-relevant genes such as caspases, cytochromes, kinases, or genes of the BCL2 family are potential candidates for a gene family investigation. Antigen families located in the MHC on human chromosome 6 are moreover a rich source for expression studies on large paralog families. Here, O2EM can be used to disclose the functionality as well as the sequence similarity within a family and the combination of both issues. Similarity of protein sequences is visualized by BLAST e-values which are translated to colours. Through this feature, the closest similar sequences can be compared with the genes similar in function. Functionality of such genes can be retraced by (coloured) expression data if respective mRNA is distinguishable.

### Proteins in functional contexts

Paralogous genes sometimes furnish protein complexes as subunits, for example *PI3K *genes (Figure 4). This special case of genes in a functional context is an interesting object for studies on gene regulation. O2EM can answer the question whether only one or all subunits are deregulated by a disturbing factor since O2EM is capable to give answers regarding co-directed differential expression of respective paralogs.

Functional context features are also covered by phylogenetic profiling [38,39], the presence or absence of proteins in organisms, which works beyond protein families and on a larger number of organisms; a corresponding approach is implemented for instance in SYSTERS [30]. O2EM complements phylogenetic profiling by elucidating the paralog populations in single protein families and extends binary presence/absence data with quantitative experimental data.

### Tissue specificity

Paralogous genes can possess different function in different tissues. After gene duplication neofunctionalization can emerge for the given gene pair and can lead to a new expression pattern of the genes across the tissues of a single organism. Hence expression profiles of orthologs may play a significant role in tissues [40] wherein orthologs with lineage-specific gene duplications tend not to have highly correlated expression profiles or even orthologs with multiple duplications show weaker correlations of expression profiles. O2EM can elucidate such traits if tissues of one organism are compared or tissue-specific samples of two species are compared.

## Conclusions

Gene expression in two organisms or individuals is, until now, compared only for the most similar genes, which is the identical gene in one organism (and two individuals) or the gene pair of closest orthologs in two different organisms. Ortho2ExpressMatrix is developed to broaden this narrow gene identity, not leaving the field of homology. Gene or protein families are visually organized into two-dimensional matrices that help users to elucidate traits of functionally connected gene family entities in two whole genomes. Future work can be expected by addition of other species, further gene family inferring approaches or even another categorization systematic: the technical background of O2EM is in principle capable to visualize gene groups beyond a categorization in families.

## Availability and requirements

O2EM is freely accessible at http://bioinf-data.charite.de/o2em/cgi-bin/o2em.pl. It is written in Perl and runs on a Linux platform using Apache2.x server. Data in the background are organized as plain text files and are available as download together with the source code for the tool (< 300 Mb). A rough database schema, the HowTo for short description and installation as well as the download link are available via the project server. The visualization of results in batches allows the installation as single-standing online application in a standard computer with adequate performance times. An interface between Laboratory Information and Management Systems or other applications and O2EM on HTTP basis is available upon request.

## List of abbreviations used

ASI: alternative splicing isoform; BLAST: Basic Local Alignment Search Tool; GEO: Gene Express Omnibus; HTTP: Hypertext Transfer Protocol; O2EM: Ortho2ExpressMatrix; PIK3CD: phosphoinositide-3-kinase, catalytic, delta polypeptide; RUNX1: runt-related transcription factor 1; URL: Uniform Resource Locator.

## Authors' contributions

TM conceived the basic idea to O2EM, he developed and implemented the tool; AHL, SK and MRS supported the development of the tool with insights from biology; RC prepared the SYSTERS version 5 data; TM, RH, MRS, and SK wrote the final manuscript. All authors read and approved the final manuscript.

## Supplementary Material

Additional file 1 **Supplementary tables and figures**. Table S1 lists the genome builts for five organisms with respective Ensembl versions integrated into Ortho2ExpressMatrix at the time of publication. Table S2 lists the implemented Microarray platforms and respective and GEO platform identifiers at the time of publication. Table S3 reports the assessment of numbers of proteins in families and protein families for pairs of organisms of four protein family inferring approaches basing on sequence data of Ensembl version 59, Table S4 reports the assessment of Ensembl 49. Figure S5 displays O2EM output examples for miRBase and TargetScan families. Figure S6 displays variations of the ratio threshold as significance criterion, which is an important O2EM selection parameter. Click here for file

## References

[B1] Domazet-Loso T Tautz D An ancient evolutionary origin of genes associated with human genetic diseases Mol Biol Evol 2008 25 12 2699 2707 10.1093/molbev/msn214 18820252 PMC2582983

[B2] Abhiman S Sonnhammer EL Large-scale prediction of function shift in protein families with a focus on enzymatic function Proteins 2005 60 4 758 768 10.1002/prot.20550 16001403

[B3] Valencia A Automatic annotation of protein function Curr Opin Struct Biol 2005 15 3 267 274 10.1016/j.sbi.2005.05.010 15922590

[B4] Krishnamurthy N Brown DP Kirshner D Sjölander K PhyloFacts: an online structural phylogenomic encyclopedia for protein functional and structural classification Genome Biol 2006 7 9 doi:10.1186/gb-2006-7-9-r83 16973001 10.1186/gb-2006-7-9-r83 PMC1794543

[B5] Brown DP Krishnamurthy N Sjölander K Automated protein subfamily identification and classification PLoS Comput Biol 2007 3 8 doi:10.1371/journal.pcbi.0030160 17708678 10.1371/journal.pcbi.0030160 PMC1950344

[B6] Liao BY Zhang J Null mutations in human and mouse orthologs frequently result in different phenotypes Proc Natl Acad Sci USA 2008 105 19 6987 6992 10.1073/pnas.0800387105 18458337 PMC2383943

[B7] Goh KI Cusick ME Valle D Childs B Vidal M Barabasi AL The human disease network Proc Natl Acad Sci USA 2007 104 21 8685 8690 10.1073/pnas.0701361104 17502601 PMC1885563

[B8] Sonnhammer EL Koonin EV Orthology, paralogy and proposed classification for paralog subtypes Trends Genet 2002 18 12 619 620 10.1016/S0168-9525(02)02793-2 12446146

[B9] Koonin EV Orthologs, paralogs, and evolutionary genomics Annu Rev Genet 2005 39 309 338 10.1146/annurev.genet.39.073003.114725 16285863

[B10] Frech C Chen N Genome-wide comparative gene family classification PLoS One 2010 5 10 e13409 10.1371/journal.pone.0013409 20976221 PMC2955529

[B11] Altschul SF Madden TL Schäffer AA Zhang J Zhang Z Miller W Lipman DJ Gapped BLAST and PSI-BLAST: a new generation of protein database search programs Nucleic Acids Res 1997 25 17 3389 3402 10.1093/nar/25.17.3389 9254694 PMC146917

[B12] Hubbard TJ Aken BL Beal K Ballester B Caccamo M Chen Y Clarke L Coates G Cunningham F Cutts T Ensembl 2007 Nucleic Acids Res 2007 35 Database D610 617 10.1093/nar/gkl996 17148474 PMC1761443

[B13] Vilella AJ Severin J Ureta-Vidal A Heng L Durbin R Birney E EnsemblCompara GeneTrees: Complete, duplication-aware phylogenetic trees in vertebrates Genome Res 2009 19 2 327 335 19029536 10.1101/gr.073585.107 PMC2652215

[B14] Tatusov RL Koonin EV Lipman DJ A genomic perspective on protein families Science 1997 278 5338 631 637 10.1126/science.278.5338.631 9381173

[B15] Krause A Stoye J Vingron M Large scale hierarchical clustering of protein sequences BMC Bioinformatics 2005 6 15 10.1186/1471-2105-6-15 15663796 PMC547898

[B16] Berglund AC Sjölund E Ostlund G Sonnhammer EL InParanoid 6: eukaryotic ortholog clusters with inparalogs Nucleic Acids Res 2008 36 Database 263 266 18055500 10.1093/nar/gkm1020 PMC2238924

[B17] Ostlund G Schmitt T Forslund K Kostler T Messina DN Roopra S Frings O Sonnhammer EL InParanoid 7: new algorithms and tools for eukaryotic orthology analysis Nucleic Acids Res 2010 38 Database D196 203 10.1093/nar/gkp931 19892828 PMC2808972

[B18] Enright AJ Kunin V Ouzounis CA Protein families and TRIBES in genome sequence space Nucleic Acids Res 2003 31 15 4632 4638 10.1093/nar/gkg495 12888524 PMC169885

[B19] Enright AJ Van Dongen S Ouzounis CA An efficient algorithm for large-scale detection of protein families Nucleic Acids Res 2002 30 7 1575 1584 10.1093/nar/30.7.1575 11917018 PMC101833

[B20] Krause A Large Scale Protein Sequence Clustering - Not Solved But Solvable Current Bioinformatics 2006 1 2 247 254 10.2174/157489306777011987

[B21] Barrett T Troup DB Wilhite SE Ledoux P Rudnev D Evangelista C Kim IF Soboleva A Tomashevsky M Marshall KA NCBI GEO: archive for high-throughput functional genomic data Nucleic Acids Res 2009 37 Database D885 890 10.1093/nar/gkn764 18940857 PMC2686538

[B22] Parkinson H Kapushesky M Kolesnikov N Rustici G Shojatalab M Abeygunawardena N Berube H Dylag M Emam I Farne A ArrayExpress update--from an archive of functional genomics experiments to the atlas of gene expression Nucleic Acids Res 2009 37 Database D868 872 10.1093/nar/gkn889 19015125 PMC2686529

[B23] Kapushesky M Emam I Holloway E Kurnosov P Zorin A Malone J Rustici G Williams E Parkinson H Brazma A Gene expression atlas at the European bioinformatics institute Nucleic Acids Res 2010 38 Database D690 698 10.1093/nar/gkp936 19906730 PMC2808905

[B24] Le HS Oltvai ZN Bar-Joseph Z Cross-species queries of large gene expression databases Bioinformatics 2010 26 19 2416 2423 10.1093/bioinformatics/btq451 20702396 PMC2944203

[B25] Flicek P Aken BL Ballester B Beal K Bragin E Brent S Chen Y Clapham P Coates G Fairley S Ensembl's 10th year Nucleic Acids Res 2010 38 Database D557 562 10.1093/nar/gkp972 19906699 PMC2808936

[B26] Haider S Ballester B Smedley D Zhang J Rice P Kasprzyk A BioMart Central Portal--unified access to biological data Nucleic Acids Res 2009 37 Web Server W23 27 10.1093/nar/gkp265 19420058 PMC2703988

[B27] The Universal Protein Resource (UniProt) in 2010 Nucleic Acids Res 2010 38 Database D142 148 19843607 10.1093/nar/gkp846 PMC2808944

[B28] Harris TW Antoshechkin I Bieri T Blasiar D Chan J Chen WJ De La Cruz N Davis P Duesbury M Fang R WormBase: a comprehensive resource for nematode research Nucleic Acids Res 2010 38 Database D463 467 10.1093/nar/gkp952 19910365 PMC2808986

[B29] Remm M Storm CE Sonnhammer EL Automatic clustering of orthologs and in-paralogs from pairwise species comparisons J Mol Biol 2001 314 5 1041 1052 10.1006/jmbi.2000.5197 11743721

[B30] Meinel T Krause A Luz H Vingron M Staub E The SYSTERS Protein Family Database in 2005 Nucleic Acids Res 2005 33 Database 226 229 D226-D229 doi: 10.1093/nar/gki030 15608183 10.1093/nar/gki030 PMC539984

[B31] Griffiths-Jones S Saini HK van Dongen S Enright AJ miRBase: tools for microRNA genomics Nucleic Acids Res 2008 36 Database 154 158 D154-D158. doi: 10.1093/nar/gkm952 17991681 10.1093/nar/gkm952 PMC2238936

[B32] Friedman RC Farh KK Burge CB Bartel DP Most mammalian mRNAs are conserved targets of microRNAs Genome Res 2009 19 1 92 105 18955434 10.1101/gr.082701.108 PMC2612969

[B33] Rivals I Personnaz L Taing L Potier MC Enrichment or depletion of a GO category within a class of genes: which test? Bioinformatics 2007 23 4 401 407 10.1093/bioinformatics/btl633 17182697

[B34] Kanehisa M Goto S Furumichi M Tanabe M Hirakawa M KEGG for representation and analysis of molecular networks involving diseases and drugs Nucleic Acids Res 2010 38 Database D355 360 10.1093/nar/gkp896 19880382 PMC2808910

[B35] Edwards H Xie C LaFiura KM Dombkowski AA Buck SA Boerner JL Taub JW Matherly LH Ge Y RUNX1 regulates phosphoinositide 3-kinase/AKT pathway: role in chemotherapy sensitivity in acute megakaryocytic leukemia Blood 2009 114 13 2744 2752 19638627 10.1182/blood-2008-09-179812 PMC2756129

[B36] Michaud J Simpson KM Escher R Buchet-Poyau K Beissbarth T Carmichael C Ritchie ME Schutz F Cannon P Liu M Integrative analysis of RUNX1 downstream pathways and target genes BMC Genomics 2008 9 363 10.1186/1471-2164-9-363 18671852 PMC2529319

[B37] Finn RD Mistry J Tate J Coggill P Heger A Pollington JE Gavin OL Gunasekaran P Ceric G Forslund K The Pfam protein families database Nucleic Acids Res 2010 38 Database D211 222 10.1093/nar/gkp985 19920124 PMC2808889

[B38] Pellegrini M Marcotte EM Thompson MJ Eisenberg D Yeates TO Assigning protein functions by comparative genome analysis: protein phylogenetic profiles Proc Natl Acad Sci USA 1999 96 8 4285 4288 10.1073/pnas.96.8.4285 10200254 PMC16324

[B39] Meinel T Daskalaki A Sequence Similarity and Function of Homologous Proteins - Phylogenetic Profiling Handbook of Research on Systems Biology Applications in Medicine 2009 1 Hershey, Pennsylvania, USA: IGI Global 143 166 10.4018/978-1-60566-076-9

[B40] Huminiecki L Wolfe KH Divergence of spatial gene expression profiles following species-specific gene duplications in human and mouse Genome Res 2004 14 10A 1870 1879 10.1101/gr.2705204 15466287 PMC524410

