# Supplementary material for: Ortho2ExpressMatrix—a web server that interprets cross-species gene expression data by gene family information
Source: BMC Genomics. 2011 Oct 4;12:483. doi: 10.1186/1471-2164-12-483 (PMC3202273; doi:10.1186/1471-2164-12-483)
Supplement: Additional file 1 — Supplementary tables and figures. Table S1 lists the genome builts for five organisms with respective Ensembl versions integrated into Ortho2ExpressMatrix at the time of publication. Table S2 lists the implemented Microarray platforms and respective and GEO platform identifiers at the time of publication. Table S3 reports the assessment of numbers of proteins in families and protein families for pairs of organisms of four protein family inferring approaches basing on sequence data of Ensembl version 59, Table S4 reports the assessment of Ensembl 49. Figure S5 displays O2EM output examples for miRBase and TargetScan families. Figure S6 displays variations of the ratio threshold as significance criterion, which is an important O2EM selection parameter. [file 1471-2164-12-483-S1.PDF]

Supplement to

Ortho2ExpressMatrix--a web server that interprets cross-species gene expression data by gene family information

Thomas Meinel [1,2,§], Michal Ruth Schweiger [2], Andreas Hanno Ludewig [3], Ramu Chenna [4], Sylvia Krobitsch [5], Ralf Herwig [2]

[1] Structural Bioinformatics Group, Institute for Physiology, Charité–University Medicine Berlin, Thielallee 71, 14195 Berlin, Germany

[2] Vertebrate Genomics Department, Max Planck Institute for Molecular Genetics, Ihnestrasse 63-73, 14195 Berlin, Germany

[3] Institute of Human Nutrition and Food Science, Christian-Albrechts-University of Kiel, Heinrich-Hecht-Platz 10, 24118 Kiel, Germany

[4] Biotechnology Center, Technical University Dresden, Tatzberg 47-49, 01307 Dresden, Germany

[5] Otto Warburg Laboratories, Max Planck Institute for Molecular Genetics, Ihnestrasse 63-73, 14195 Berlin, Germany

[§]Corresponding author

Email address:

TM: thomas.meinel@charite.de

Table S1

Genome builts for five organisms with respective Ensembl versions integrated into Ortho2ExpressMatrix at the time of publication.

Species	Ensembl v 59	Ensembl v 53	Ensembl v 49
Hs	GRCh37	NCBI36	NCBI36
Mm	NCBIM37	NCBIM37	NCBIM37
Ce	WS210	WS190	WS180
Dm	BDGP5.13	BDGP5.4	BDGP5.4
Sc	SGD1.01	SGD1.01	SGD1.01

Table S2

Microarray platforms and respective and GEO platform identifiers (GPL) implemented in Ortho2ExpressMatrix at the time of publication.

Microarray platform	GEO GPL	Ensembl version in O2EM
Affymetrix C elegans	GPL200	49, 53, 59
Affymetrix Drosophila 2	GPL1322	53, 59
Affymetrix MG U74a 2	GPL81	49, 53, 59
Affymetrix Moe 430a	GPL339	49, 53, 59
Affymetrix Moe 430b	GPL340	49, 53, 59
Affymetrix Mouse430 2	GPL1261	49, 53, 59
Affymetrix Mouse430a 2	GPL8321	49, 53, 59
Affymetrix U133A	GPL96	49, 53, 59
Affymetrix U133A2	GPL571	49, 53, 59
Affymetrix U133B	GPL97	49, 53, 59
Affymetrix U133Plus2	GPL570	49, 53, 59
Affymetrix U95A	GPL91	49, 53, 59
Affymetrix U95Av2	GPL8300	49, 53, 59
Affymetrix Yeast 2	GPL2529	49, 53, 59
Agilent CGH 44b	GPL2879	59
Agilent Hs miRNA_V1_016436	GPL8617	59
Agilent Hs miRNA_V2_019118	GPL8227	59
Agilent Hs miRNA_V3_021827	GPL10850	59
Agilent Mm miRNA_V1_019119	GPL8824	59
Agilent OligoArray	GPL2875	59
Agilent WG	GPL4134	59
Agilent WholeGenome	GPL4133	59
Agilent WholeGenome (alternative annotation)	GPL6480	59
Illumina Mouse Ref-6 V1	GPL6333	49, 53
Illumina MouseRef-8 v2.0	GPL6885	53
Illumina MouseWG-6 v2.0	GPL6887	59
Illumina WG 6 v1	GPL6097	59
Illumina WG 6 v2	GPL6102	59
Illumina WG 6 v3	GPL6884	59
Illumina mouse-6 v1.1	GPL6105	59
Illumina v1	GPL5060	49, 53
Illumina v2	GPL6790	49, 53
Wustl C elegans	GPL5883	49

Table S3

Numbers of proteins in families and protein families for pairs of organisms of four protein family inferring approaches, basing on sequence data of Ensembl version 59, Aug 2010: EnsemblCompara, InParanoid7, SYSTERS, and Ensembl Family. Included organisms are human (Hs), mouse (Mm), worm (Ce), fruit fly (Dm) and yeast (Sc). The five columns on the left – proteins in families – should be read: There are 67304 human protein sequences along with 43953 mouse sequences in human-mouse families of the EnsemblCompara approach; those sequences are grouped into 16552 families. Numbers of respective protein families are given in the five columns to the right. Gray cells: paralogous sequences or families (in one organism). Such numbers are not available for InParanoid due to the methodology. EnsemblCompara and InParanoid families are generated for pairs of organisms whereas for SYSTERS and Ensembl Family, those clusters comprise similar sequences independent from organisms, pairwise data extractions are made for comparability. The total number of reference protein sequences can be found in the Ensembl Family sub-matrix diagonal (yellow cells) due to the fact that the Ensembl Family approach generally categorizes all proteins of the existing sequence space into a family. The lower two sections comprise microRNA families of miRBase and TargetScan made in an analogous process; there are no microRNAs for Sc.

Table S3 (continued)

Ensembl 59	proteins in families					protein families				
	Hs	Mm	Ce	Dm	Sc	Hs	Mm	Ce	Dm	Sc
EnsemblCompara – Ensembl version 59 (BioMart)										
Hs	32703	67304	37347	40349	19546	3750	16552	4797	5728	2167
Mm	43953	23630	23868	25830	11867		3821	4794	5736	2169
Ce	11288	11256	13009	11270	6422			2608	4672	2023
Dm	12815	12828	11894	8637	6474				2021	2077
Sc	2540	2545	2387	2452	1302					744
InParanoid7 – standardized on Ensembl protein IDs, Ensembl version 59										
Hs		17436	8105	8728	4090		16376	4562	5527	2154
Mm	17446		9434	10086	4201			4601	5557	2171
Ce	5585	5689		5122	2610				4200	1819
Dm	6355	6407	5191		2830					1977
Sc	2534	2548	2191	2371						
SYSTERS4 – standardized on Ensembl protein IDs, Ensembl version 59										
Hs	26705	24090	14444	16062	8081	7516	5873	2768	3358	1415
Mm	21439	24041	12384	13901	6582		7732	2662	3225	1369
Ce	8880	8763	21829	8855	4948			9284	2711	1350
Dm	11941	11658	10646	19012	6023				7310	1390
Sc	2481	2418	2437	2468	6266					4422
SYSTERS5 – standardized on Ensembl protein IDs, Ensembl version 59										
Hs	30484	24941	11910	14656	4668	7853	5341	2072	2703	724
Mm	19829	23552	8165	10404	2951		7515	1832	2367	653
Ce	5873	5429	16111	5400	2286			6460	1830	653
Dm	8591	7939	6416	15551	2405				6431	657
Sc	1067	990	994	986	3260					2505
Ensembl Family – Ensembl version 59 (BioMart)										
Hs	74579	67036	20796	26359	6796	14168	10997	2367	3077	875
Mm	44429	50957	13025	16698	3936		14836	2367	3076	874
Ce	6531	6542	27975	6104	2323			14974	2175	793
Dm	8255	8238	6326	21899	2246				11385	833
Sc	1139	1138	1052	1092	6696					5724

Ensembl 59	microRNAs in families					microRNA families				
	Hs	Mm	Ce	Dm	Sc	Hs	Mm	Ce	Dm	Sc
miRBase 16 – standardized on miRBase mature IDs										
Hs	919	555	31	95	-	443	203	5	23	-
Mm	698	809	38	112	-		253	5	23	-
Ce	8	8	151	16	-			96	10	-
Dm	30	30	19	139	-				114	-
Sc	-	-	-	-	-	-	-	-	-	-
TargetScan 5.1 – standardized on miRBase mature IDs										
Hs	677	311	0	9	-	545	211	0	7	-
Mm	303	491	0	9	-		373	0	7	-
Ce	0	0	113	11	-			68	5	-
Dm	10	10	5	146	-				121	-
Sc	-	-	-	-	-					-

Table S4

See information as in Table S3, however with InParanoid6 and without microRNA families. Numbers should be read as in Table S3.

Ensembl 49	proteins in families					protein families				
	Hs	Mm	Ce	Dm	Sc	Hs	Mm	Ce	Dm	Sc
EnsemblCompara – Ensembl version 49 (BioMart)										
Hs	19158	38520	16357	18843	9262	3568	16584	4202	5143	2105
Mm	32740	17766	13845	15868	7720		3546	4199	5152	2104
Ce	8758	8762	7716	8360	4916			2124	3978	1922
Dm	10003	10008	8251	4875	4875				1452	1977
Sc	2505	2505	2287	2331	1172					681
InParanoid6 – standardized on Ensembl protein IDs, Ensembl version 49										
Hs		26807	8900	9516	2573		15559	4658	5586	2165
Mm	16518		7539	6287	2560			4614	5503	2166
Ce	7698	14415		5288	2302				4325	1891
Dm	6351	14486	7144		2414					1996
Sc	4298	6309	3620	2913						
SYSTERS4 – standardized on Ensembl protein IDs, Ensembl version 49										
Hs	25243	22013	13119	14665	7232	8589	6203	2839	3467	1436
Mm	19683	22131	11077	12423	5920		8171	2711	3302	1391
Ce	9072	8906	22063	8940	5004			9413	2729	1351
Dm	11224	10944	9877	17918	5635				7442	1396
Sc	2512	2447	2442	2477	6284					4425
SYSTERS5 – standardized on Ensembl protein IDs, Ensembl version 49										
Hs	26541	21751	10008	12332	3784	8584	5665	2122	2724	745
Mm	19271	22543	7794	9808	2791		7810	1871	2385	665
Ce	6047	5602	16370	5453	2338			6568	1835	658
Dm	7878	7285	5883	14123	2220				6282	652
Sc	1093	1006	997	977	3268					2508
Ensembl Family – Ensembl version 49 (BioMart)										
Hs	45855	42809	14886	16308	2637	2507	2075	661	770	248
Mm	36022	39667	12353	12371	2380		2443	652	768	244
Ce	10609	10660	26902	8986	1371			1753	588	214
Dm	10710	10201	10405	20815	951				995	218
Sc	2586	2225	2638	1756	6698					434

Figure S5

(A)

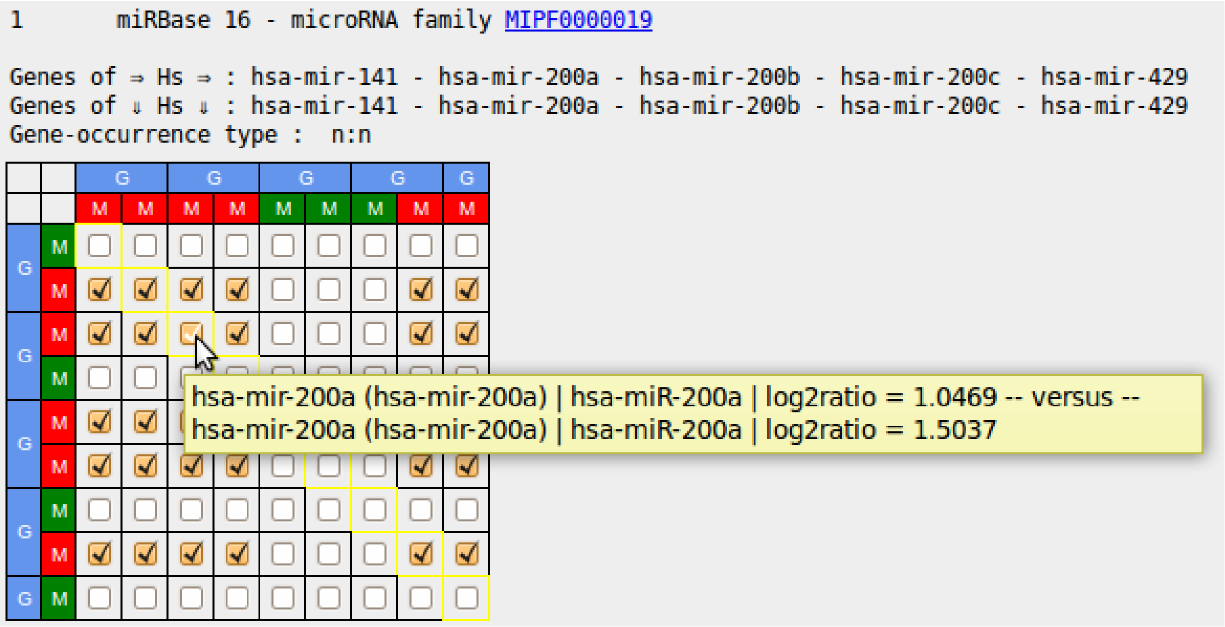

(B)

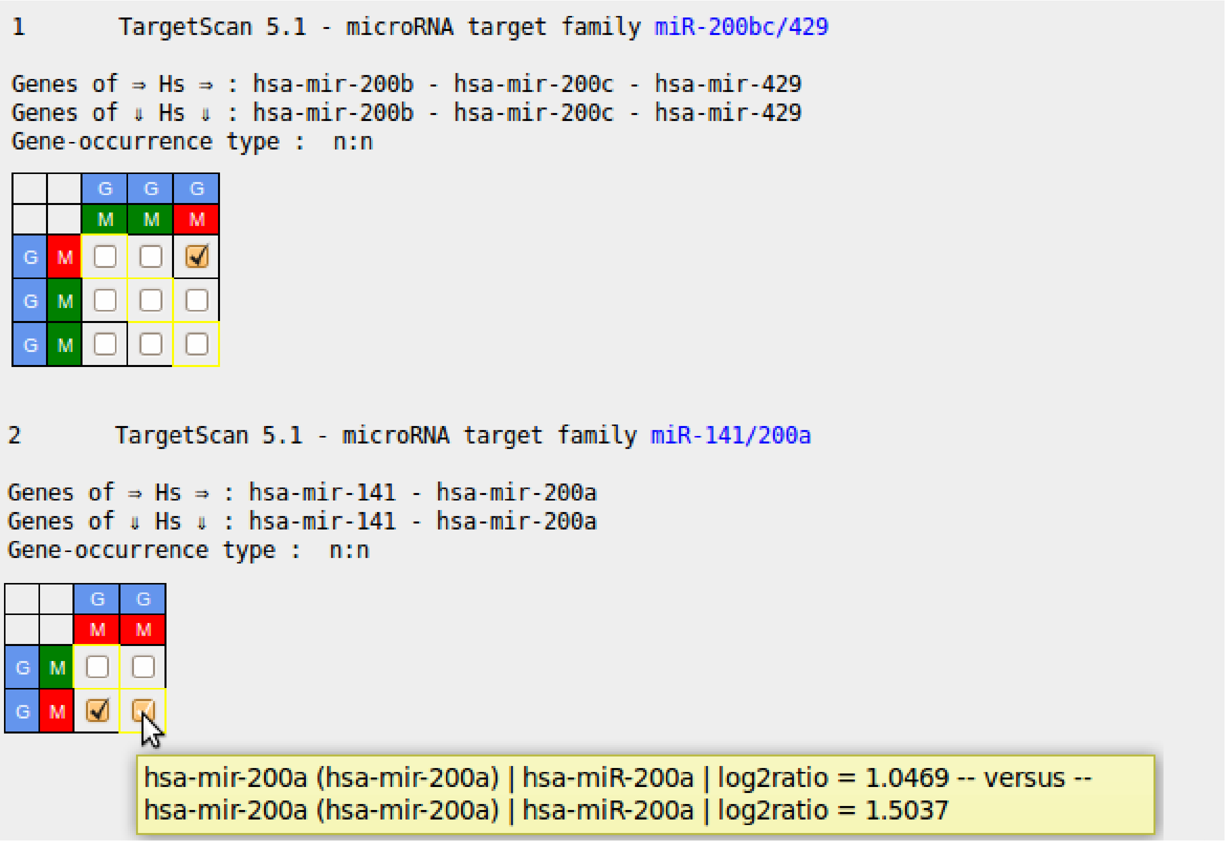

Ortho2ExpressMatrix screenshots for a particular microRNA family: (A) The miRBase family MIPF0000019 consists of five mature microRNAs (star-microRNAs are added to the family in O2EM). Therefore, the hairpin microRNA (mouse-over blue cells, 'G' icons, provide links to miRBase) is occasionally associated with the forward-stranded and the star-microRNA (mouse-over colored cells 'M' provides information on microRNA species and respective log2-ratio). Differential expression data (values

are translated to colors, red/black/green) are calculated from random gene expression data as provided in the test file of the online tool. HTML boxes are pre-selected as automatically checked-in for activated genes; mouse-over shows the pair of mature microRNAs and both log2-ratios.

(B) Identical five hairpin microRNAs as in (A), but grouped according to TargetScan families miR-200bc/429 and miR-141/200a; such families are target-oriented. The link behind the family name leads to scored target genes in TargetScan. Star-microRNAs are not included in TargetScan families.

Figure S6

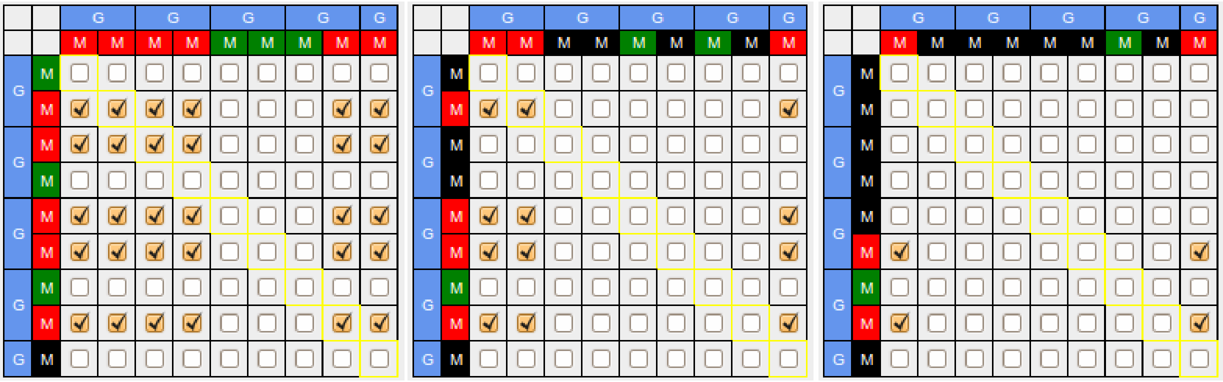

Variation of the significance criterion, an important selection parameter in O2EM - Three further significance thresholds are applied to the default setting in the O2EM web tool and presented in Figure S5A (0.75 < ratio < 1.33 (-0.41 < log2-ratio < 0.41): Left: 0.50 < ratio < 2.00 (-1 < log2-ratio < 1); Middle: 0.25 < ratio < 4 (-2 < log2-ratio < 2); Right: 0.125 < ratio < 8 (-3 < log2-ratio < 3). The increase of non-significant differential expressed microRNAs is represented in the increase of black gene icons ‘M’ and in the decrease of the number of automatically checked HTML boxes.
